# Risk of hepatitis B reactivation in HBsAg−/HBcAb+ patients after biologic or JAK inhibitor therapy for rheumatoid arthritis: A meta‐analysis

**DOI:** 10.1002/iid3.780

**Published:** 2023-02-09

**Authors:** Xuezhi Hong, Yanhua Xiao, Liyan Xu, Lei Liu, Hailu Mo, Hanyou Mo

**Affiliations:** ^1^ Department of Rheumatology and Immunology The First Affiliated Hospital of Guilin Medical University Guilin People's Republic of China; ^2^ Tianjin hospital Tianjin China

**Keywords:** biologic therapy, hepatitis B virus, reactivation, rheumatoid arthritis

## Abstract

**Background:**

The risk of hepatitis B virus (HBV) reactivation after biologic and targeted synthetic disease‐modifying antirheumatic drugs (b/tsDMARDs) therapy in patients with rheumatoid arthritis (RA) combined with HBsAg–/HBcAb+ is still inconsistent.

**Methods:**

We conducted a systematic review of existing databases from 1977 to August 22, 2021. Studies of RA patients combined with HBsAg−/HBcAb +, treated with b/tsDMARDs and the reported number of HBV reactivation were included.

**Results:**

We included 26 studies of 2252 HBsAg−/HBcAb+ RA patients treated with b/tsDMARDs. The pooled HBV reactivation rate was 2.0% (95% confidence interval [CI]: 0.01−0.04; *I* ^2^ = 66%, *p* < .01). In the subgroup analysis, the HBV reactivation rate of rituximab (RTX), abatacept, and inhibitors of Janus kinase (JAK), interleukin‐6 (IL‐6), and tumor necrosis factor‐α (TNF‐α) were 9.0% (95% CI: 0.04−0.15; *I* ^2^ = 61%, *p* = .03), 6.0% (95% CI: 0.01−0.13; *I* ^2^ = 40%, *p* = .19), 1.0% (95% CI: 0.00−0.03; *I* ^2^ = 41%, *p* = .19), 0.0% (95% CI: 0.00−0.02; *I* ^2^ = 0%, *p* = .43), 0.0% (95% CI: 0.00−0.01; *I* ^2^ = 0%, *p* = .87), respectively. While HBsAb‐ patients have a significant risk of reactivation (odds ratio [OR] = 4.56, 95% CI = 2.45−8.48; *I* ^2^ = 7%, *p* = .37), low HBsAb+ group also display a significant risk of reactivation (OR = 5.45, 95% CI: 1.35−21.94; *I* ^2^ = 0%, *p* = .46).

**Conclusions:**

This meta‐analysis demonstrates the highest potential risk of HBV reactivation in HBsAg−/HBcAb+ RA patients receiving RTX treatment, especially HBsAb− patients. Our study furthers the understanding of the prophylactic use of anti‐HBV drugs in such patients. However, it is relative safety to use the inhibitors of IL‐6, TNF‐α, and JAK in these patients.

## Graphical abstract

Abatacept is second to rituximab (RTX) at high risk of HBV reactivation in RA patients combined with HBsAg−/HBcAb + . Prophylactic use of anti‐HBV drugs should also be considered in HBsAg−/HBcAb+ RA patients treated with abatacept if they have multiple risk factors.

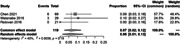

## 1 INTRODUCTION

Rheumatoid arthritis (RA) is a common connective tissue disease which can cause bone and cartilage destruction and affects approximately 1% of the global population.^1^ With the development of biomedical technology over the past two decades, there are now a number of treatment strategies to control RA progression, such as glucocorticoids, methotrexate, biological and targeted synthetic disease‐modifying antirheumatic drugs (b/tsDMARDs) such as antitumor necrosis factor‐α (TNF‐α), rituximab (RTX), abatacept, and tofacitinib.^2^ With the prolonged use of b/tsDMARDs agents, increased attention has been paid to its adverse effects such as opportunistic infections, herpes zoster, tuberculosis, and hepatitis B or C.^3^

Hepatitis B virus (HBV) infection is a global problem. World Health Organization (WHO) estimates that there are 296 million people suffering from chronic HBV and nearly 820,000 patients died owing to liver cirrhosis or carcinoma in 2019 alone.^4^ HBV carriers run a significant risk of reactivation when using b/tsDMARDs, and hence are advised to have prophylactic treatment with antiviral drugs.^5^ Patients with resolved HBV infection (HBsAg‐/HBcAb +) also have a risk of HBV reactivation. However, this reactivation rate varied considerably in different studies, ranging from 0% to 21.43%,^1^, ^6^ even in a small HBsAb‐ group (only seven patients), the abatacept reactivation can even up to 28.6%.^1^ These variations can be attributed to sample size, length of follow‐up, types of immunosuppression agents, and clinical characteristics of the patients.

Therefore, we conducted a systemic review and meta‐analysis of the most recent studies to estimate the impact of various b/tsDMARDs therapies on the risk of HBV reactivation in HBsAg−/HBcAb+ RA patients and enable rheumatologists to administer timely prophylactic antiviral treatment to patients who are more likely to experience a relapse.

## 2 MATERIALS AND METHODS

### 2.1 Search strategy

The study was conducted in accordance with the PRISMA guidelines.^7^ We used the MeSH terms “Hepatitis B virus,” “HBV” or “Hepatitis B” in combination with “Rheumatoid arthritis” or “RA” to search the Cochrane Library database (up to August 22, 2021), PubMed (up to August 22, 2021), Chinese National Knowledge Infrastructure (up to August 22, 2021), and Wanfang databases (up to August 22, 2021). After reading the complete text and discovering the pertinent literatures, manual searches were further conducted.

### 2.2 Study selection and data extraction

Two investigators (L.‐Y. X. and L. L.) independently screened the studies for eligibility based on the inclusion/exclusion criteria. Among the authors, disagreements were resolved by discussion. Inclusion criteria: (a) patients diagnosed with RA and HBsAg−/HBcAb + ; (b) treatment with b/tsDMARDs drugs or in combination with glucocorticoid (GC) agents; (c) description of HBV reactivation; (d) clinical or observational studies. Case reports, review, meta‐analysis, conference papers, less than 10 patients, and animal models were excluded. HBV reactivation was defined as the reappearance of HBsAg, a ≥ 10‐fold increase in HBV DNA level from baseline, or switch in HBV DNA detection from a negative to a positive.

A data collection form was used to collect data from all included studies by two authors (L. L. and H.‐L. M.). The extracted data include: (a) study characteristics (author, year, country, and design); (b) patient's information (characteristics of hepatitis, sample size, sex, and age); (c) intervention and comparison (drug agents, sample size, and follow‐up time); (d) the quantity of HBV reactivation. Missing or unclear information is not be included in the final analysis.

### 2.3 Statistical analysis

The pooled reactivation rate of HBV was estimated by the fixed effect model or the random effects model based on heterogeneity among studies. Considering the variety of countries in the selected articles for meta‐analyses, random effects model was used in our meta‐analysis. Freeman−Tukey double arcsine transformation was used to calculate pooled estimates of proportions with 95% confidence intervals (CI). The DerSimonian and Laird (D−L) random‐effects model was applied to dichotomous data with respect to the calculation of the overall odds ratio (OR) and 95% CI. We assessed bias using a funnel chart: if the funnel chart was asymmetrical, bias was considered to exist. Correlation analysis was performed by Spearman correlation. Data analysis was performed using R language version 4.1.0.

## 3 RESULTS

### 3.1 Study description

**Figure 1:**
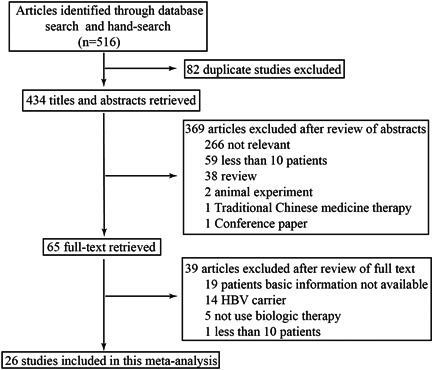
Flowchart of article selection.

The selection process is shown in Figure 1. A total of 26 studies met our inclusion criteria and involved 2252 RA patients with HBsAg−/HBcAb + . The duration of follow‐up time ranged from 3 to 75 months and the reactivation rate of HBV ranged from 0% to 16.13%. All studies were published between 2009 and 2021. There were 6, 3, 2, 4, and 12 articles describing RTX, abatacept, and inhibitors of Janus kinase (JAK), IL‐6, and TNF‐α in detail, respectively. Twelve out of 26 articles also described the incidence of HBV reactivation in HBsAb− and HBsAb+ patients. Countries (number) of the studies were as follows: China (9),^1^, ^3^, ^6^, ^8^, ^9^, ^10^, ^11^, ^12^, ^13^ Italy (7),^14^, ^15^, ^16^, ^17^, ^18^, ^19^, ^20^ Japan (6),^2^, ^21^, ^22^, ^23^, ^24^, ^25^ Korea (1),^26^ Greece (1),^27^ France (1),^28^ and Multi‐national (1).^29^ Table 1 displays the basic characteristics of the 26 studies in our meta‐analysis.

**Table 1:** Studies with HBV reactivation among HBsAg‐/HBcAb+ patients after biologic or JAK inhibitor therapy for RA.

References			Total	Design	Event		Biologic agents					
	HBV re	Country				Ratio	TNF‐α	CD20	CTLA‐4	IL‐6	JAK	Follow‐up
	definition	/Origin				（%）	n.(re)	n.(re)	n.(re)	n.(re)	n.(re)	(months)
Chen et al.^1^	1	Taiwan	489	Ret	27	5.52	255(3)	84(18)	69(6)	81(0)		75
Watanabe et al.^2^	2/3	Japan	59	Ret	5	8.48	‐					41
Kuo et al.^3^	1/3	Taiwan	50	Ret	4	8		50(4)				46.8
Harigai et al.^29^	2/3	MC	215	pro	4	1.86					215(4)	15.5
Ditto et al.^14^	3	Italy	112	Ret	10	8.93	‐					‐
Tien et al.^8^	1	Taiwan	272	pro	2	0.74	‐					‐
Watanabe et al.^21^	3	Japan	152	Ret	7	4.61	98(3)		29(3)	25(1)		15
Chen et al.^9^	1/2	Taiwan	103	Ret	9	8.74		103(9)				61
Chen et al.^6^	2	Taiwan	75	Ret	0	0					75(0)	‐
Ahn et al.^26^	1/3	korea	15	Ret	0	0				15(0)		9.4
Tien et al.^10^	1/2/3	Taiwan	44	Ret	4	9.09		44(4)				25.4
Chen et al.^11^	1/2/3	China	41	Pro	0	0				41(0)		3
Papalopoulos et al.^27^	2/3	Greece	128	Ret	2	1.56	69(0)	‐				24
Varisco et al.^15^	1/3	Italy	33	Ret	1	3.03		33(1)				34
Padovan et al. 2016^16^	1	Italy	21	Ret	0	0			21(0)			24
Nakamura et al.^22^	2	Japan	57	Ret	3	5.26	‐					18
Jin et al.^12^	2/3	China	10	Pro	0	0	10(0)					12
Barone et al.^17^	1	Italy	58	Pro	0	0	‐					‐
Ballanti et al.^18^	1/2	Italy	25	Ret	0	0	26(0)					72
Biondo et al.^19^	1/3	Italy	12	Pro	0	0	12(0)					45
Urata et al.^23^	1/3	Japan	62	Pro	10	16.13	‐					18
Tamori et al.^24^	2	Japan	42	Pro	0	0	42(0)					24
Mori et al.^25^	3	Japan	36	Pro	1	2.78	31(1)			5(0)		12
Lan et al.^13^	1/2	Taiwan	70	Pro	1	1.43	70(1)					12
Caporali et al.^20^	1/3	Italy	59	Pro	0	0	59(0)					42.52
Charpin et al.^28^	1/3	France	12	Pro	0	0	12(0)					‐

*Note*: HBV reactivation was defined as the reappearance of HBsAg (1), a ≥ 10‐fold increase in HBV DNA level from baseline (2), or switch in HBV DNA detection from a negative to a positive (3).

Abbreviations: IL‐6, interleukin‐6; MC, multicountry; n. (re), number of patients using the respective drugs (number of hepatitis B reactivation); pro, prospective; RA, rheumatoid arthritis; ratio (%), percentage of reactivation; ret, retrospective; TNF‐α, tumor necrosis factor‐α; ‐, unknown.

### 3.2 HBV reactivation in different b/tsDMARDs therapy

**Figure 2:**
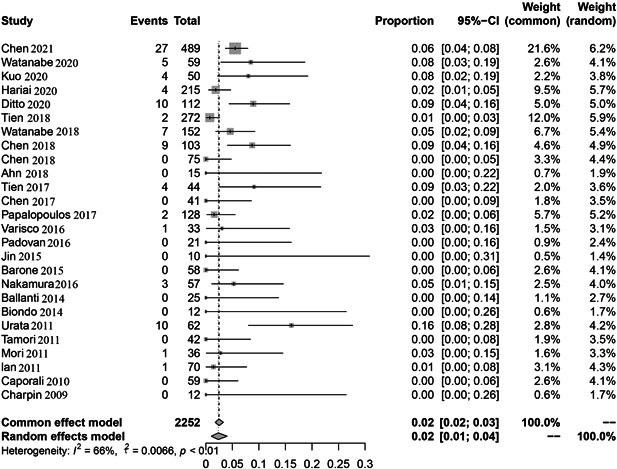
The pooled rate of Hepatitis B virus (HBV) reactivation among HBsAg‐/HBcAb+ patients after biologic or JAK inhibitor therapy for rheumatoid arthritis (RA).

In our literature studies, the reactivation rate ranged from 0% to 16.13%. Owing to significant heterogeneity (*I* ^2^ = 66%, *p* < .01), random effects model was used. The pooled HBV reactivation rate was 2.0% (90 reactivations in 2252 patients) as seen in Figure 2.

**Figure 3:**
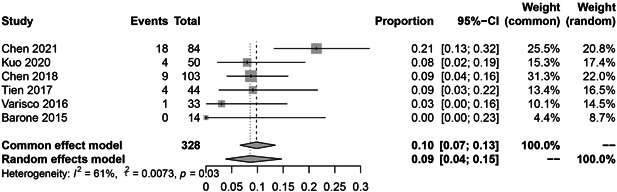
The pooled rate of Hepatitis B virus (HBV) reactivation among HBsAg‐/HBcAb+ patients after rituximab therapy for rheumatoid arthritis (RA).

**Figure 4:**
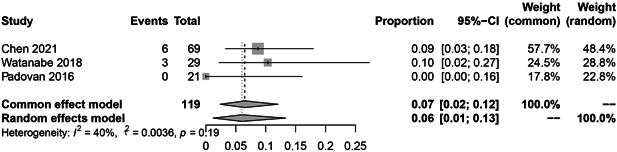
The pooled rate of Hepatitis B virus (HBV) reactivation among HBsAg‐/HBcAb+ patients after abatacept therapy for rheumatoid arthritis (RA).

**Figure 5:**
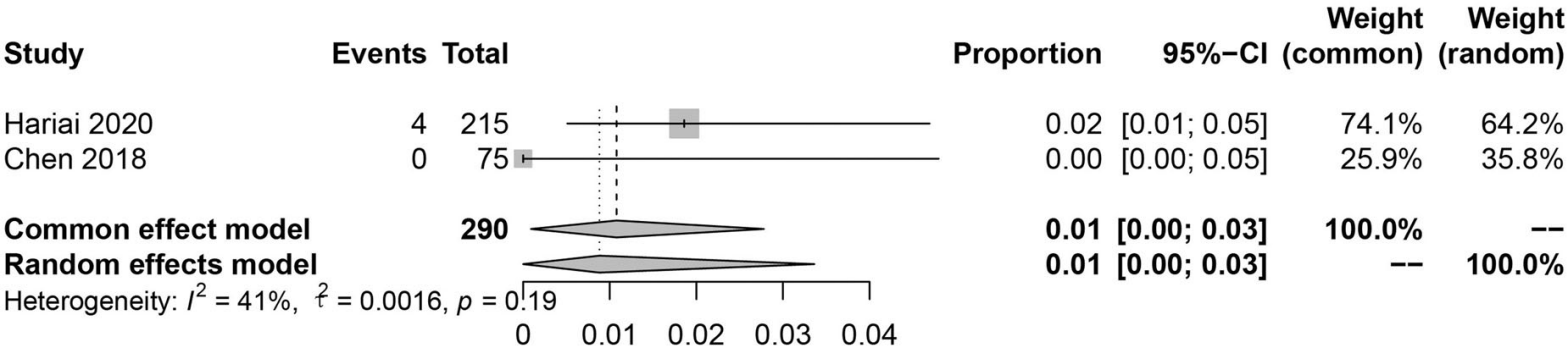
The pooled rate of Hepatitis B virus (HBV) reactivation among HBsAg‐/HBcAb+ patients after JAK inhibitor therapy for rheumatoid arthritis (RA).

**Figure 6:**
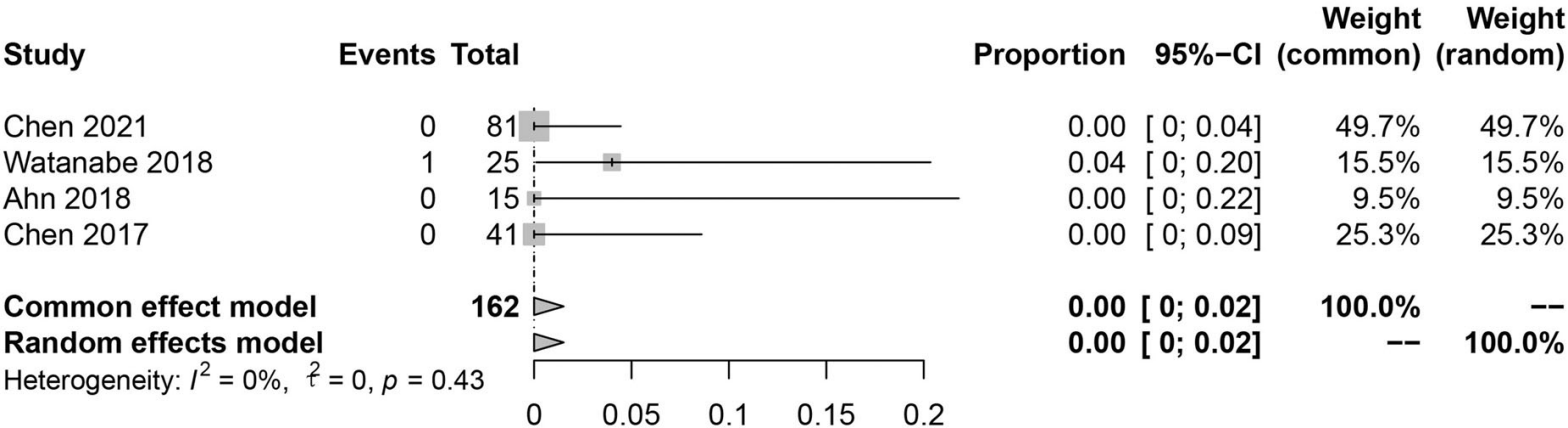
The pooled rate of hepatitis B virus (HBV) reactivation among HBsAg‐/HBcAb+ patients after interleukin‐6 (IL‐6) inhibitor therapy for rheumatoid arthritis (RA).

**Figure 7:**
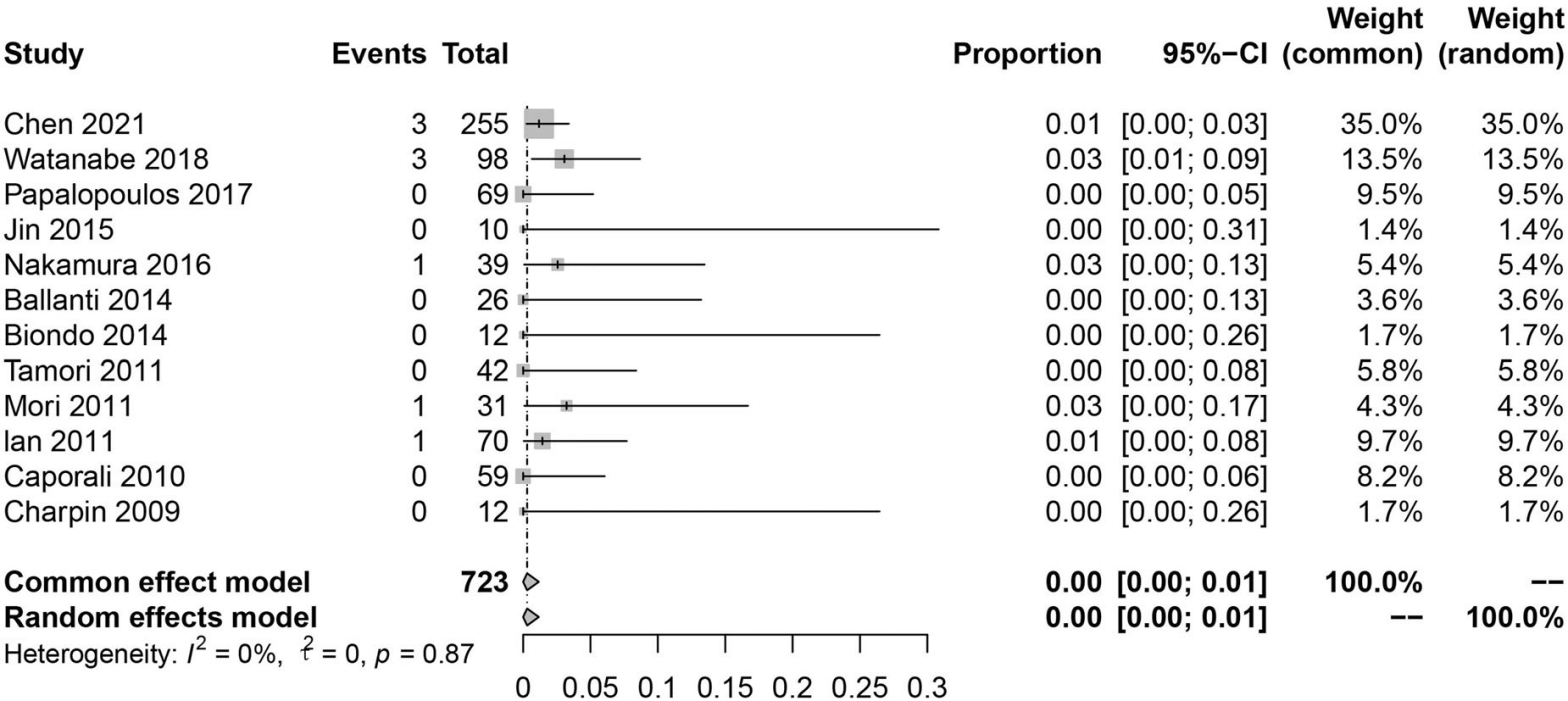
The pooled rate of hepatitis B virus (HBV) reactivation among HBsAg‐/HBcAb+ patients after tumor necrosis factor‐α (TNF‐α) inhibitor therapy for rheumatoid arthritis (RA).

In the subgroup analysis, the HBV reactivation rate of RTX, abatacept, and inhibitors of JAK, IL‐6, and TNF‐α were 9.0% (36 reactivations in 328 patients; Figure 3; *I* ^2^ = 61%, *p* = .03), 6.0% (9 reactivations in 119 patients; Figure 4; *I* ^2^ = 40%, *p* = .19), 1.0% (4 reactivations in 290 patients; Figure 5; *I* ^2^ = 41%, *p* = .19), 0.0% (1 reactivation in 162 patients; Figure 6; *I* ^2^ = 0%, *p* = .43), 0.0% (12 reactivations in 723 patients; Figure 7; *I* ^2^ = 0%, *p* = .87), respectively.

### 3.3 HBV reactivation in HBsAb− and HBsAb + patients

**Figure 8:**
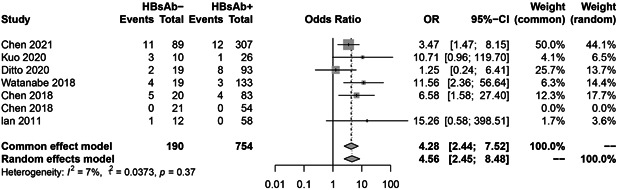
The pooled risk of hepatitis B virus (HBV) reactivation in HBsAb− group compared with HBsAb+ group.

**Figure 9:**
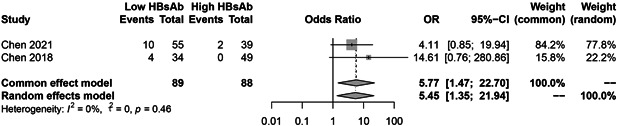
Hepatitis B virus (HBV) reactivation in low and high titer of HBsAb+ patients.

Figure 8 illustrates the pooled risk of HBV reactivation in HBsAb− group compared with HBsAb+ group. HBsAb− patients have a significant risk of reactivation (OR = 4.56, 95% CI = 2.45−8.48). We further analyzed the risk of reactivation of HBV in the low HBsAb + (HBsAb < 100 mIU/mL) and high HBsAb + (HBsAb ≥100 mIU/mL) groups. Our results showed that low HBsAb+ group have a significant risk of reactivation (OR = 5.45, 95% CI = 1.35−21.94) (Figure 9).

### 3.4 HBV reactivation in therapy groups with/without glucocorticoid (GC)

**Figure 10:**
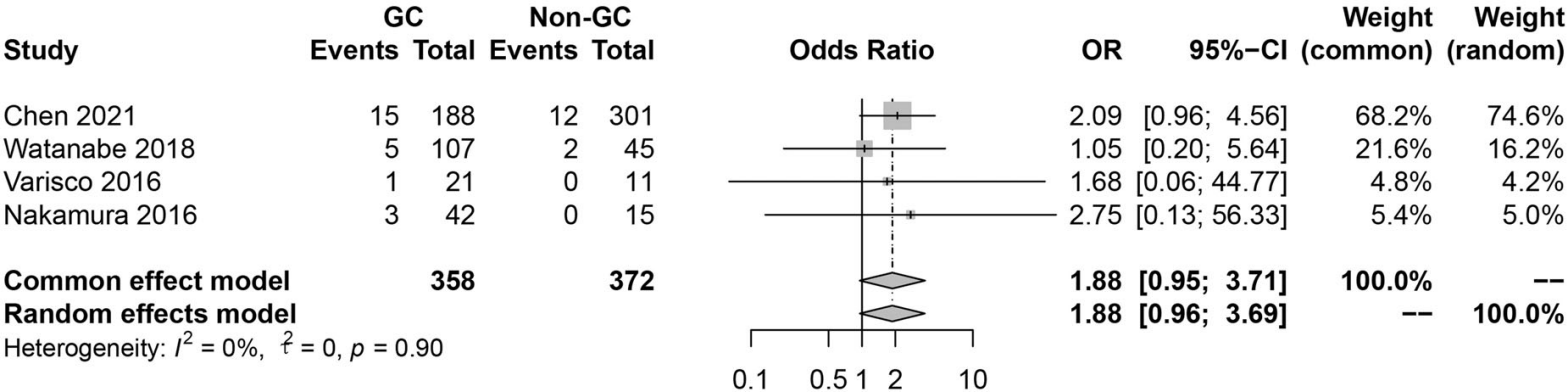
Hepatitis B virus (HBV) reactivation in therapy groups with/without glucocorticoid (GC).

Figure 10 illustrates the pooled risk of HBV reactivation in the GC group compared with the non‐GC group. GC group patients have a higher risk of reactivation (OR = 1.88, 95% CI = 0.96−3.69).

### 3.5 Risk of bias within studies and correlation analysis

Since all of the funnel plots were symmetric, there was no discernible publishing bias (Supporting Information: Figure 1). The correlation between follow‐up time and HBV reactivation was not found in different biologic agents. All the plots are depicted in Supporting Information: Figure 2.

## 4 DISCUSSION

Although the incidence reported in the literature was highly variable,^1^, ^6^ there is a possible risk of b/tsDMARDs treatment‐induced HBV reactivation in RA patients who are HBsAg−/HBcAb + .^1^, ^6^ For instance, de novo hepatitis in patients with resolved HBV can cause fulminant hepatitis and, in some cases, even death.^6^ The question of whether preventive anti‐HBV medication should be provided to patients with resolved HBV infection before using b/tsDMARDs therapies is attracting increased attention.

Our study showed that the pooled reactivation rate of HBV in RTX group is 9%. Its reactivation is mainly attributed to RTX which can deplete pre‐B and mature B cells. A retrospective study showed that HBsAb titers reduced from 296.0 ± 417.3 mIU/mL to 187.0 ±332.5 mIU/mL after RTX therapy.^9^ B‐cell depletion may break CD8 + cytotoxic T cell and alter T‐lymphocyte activity.^30^ Furthermore, a 75‐month follow‐up research with a 21.43% reactivation rate serves as a reminder that even after RTX administration is discontinued, patients are still at heightened risk. Antiviral therapy is therefore advised for HBsAg‐/HBcAb+ RA patients who have been prescribed RTX. Our results are consistent with the recommendations of the European Association for the Study of the Liver (EASL)^31^ and the American Association for the Study of Liver Disease (AASLD).^32^ In the EASL, RTX was identified to induce a high risk of HBV reactivation and advises antiviral treatment. Likewise, AASLD proposes an antiviral therapy for at least 12 months after completion of RTX therapy. Nevertheless, our investigations suggest that despite a 12‐month antiviral treatment, HBsAg‐/HBcAb+ patients still risk the possibility of relapsing. Thus, additional prospective research is still required to ascertain how long an antiviral therapy is necessary following the completion of RTX treatment.

According to the analysis, abatacept is only second to RTX at inducing a risk hepatitis B reactivation in RA patients. HBV has a pooled reactivation ratio of 7%. As abatacept is a blocker for T‐cell activation, it can abrogate CD4 + and CD8 + T cells specific for HBV.^1^ While three articles reported reactivation rate of HBV (10.34% as the highest), no association between reactivation and follow‐up time was determined. Although abatacept has a moderate risk of reactivating HBV, it is not included in antiviral therapy as per EASL guidance. However, one publication (which included just seven patients) claimed that the reactivation rate in the HBsAb‐ group could potentially reach 28.6%.^1^ According to the ranking for the risk of HBV reactivation across chemotherapeutic agents and immunosuppressants from the American Gastroenterological Association (AGA) Institute,^33^, ^34^ the risk of HBV reactivation with abatacept is moderate, antiviral therapy should be taken into consideration when abatacept is administered to patients with comorbidities, such as HBsAb‐, GC, old age, and the use of other immunosuppressive agents.

The pooled reactivation rates of the inhibitors of JAK, IL‐6, and TNF‐α were low at 1%, 0%, and 0%, respectively. However, according to some articles, their relapse rate can reach 1.86%, 4%, and 3.23%.^8^, ^21^, ^25^ Our pooled results suggest that all three inhibitors are quite safe for HBsAg‐/HBcAb+ RA patients. Hence, these b/tsDMARDs agents should be considered primarily when such patients require a b/tsDMARDs treatment or switch to another therapy strategy to manage their disease, owing to their low reactivation rate and cost‐effectiveness. Antiviral therapy may not be advised as concomitant therapy when they were used. Frequent monitoring of ALT, HBsAg, and HBV DNA were also recommended in AASLD guidance for these patients.

Patients with HBsAb− or lower titers of anti‐HBs, old age, past history of hepatitis, and use of glucocorticoid are risk factors for HBV reactivation.^1^, ^35^ Due to the limitations of the included literatures, only some of the literature reported relapse in patients with HBsAb− or HBsAb + , high or low titer of HBsAb+ and with or without GC using were obtained. From our meta‐analysis results it can be deduced that the risk of reactivation is 4.16 times higher in HBsAb− patients than HBsAb+ patients, 5.45 times higher in low HBsAb+ group than high HBsAb+ group and 1.88 times higher in GC group than non‐GC group. In Chen study, the reactivation ratio can even reach 25% in HBsAb− patients,^6^ and based on the AGA report, HBsAg‐/HBcAb+ patients daily treated with high‐dose (>20 mg prednisone or equivalent)/moderate‐dose (10−20 mg prednisone or equivalent) corticosteroids for ≥ 4 weeks, developed moderate risk and patients administered with low‐dose (<10 mg prednisone or equivalent) corticosteroids for ≥ 4 weeks developed low risk of HBV reactivation.^33^, ^34^ Therefore, concerning HBsAb− patients combined with other risk reactivation factors, prophylactic hepatitis B vaccine or antiviral therapy should be given before starting biologic treatment.

There are several limitations in our study. First, the studies included were mainly from regions with a high prevalence of hepatitis B, such as China and Japan. Very few literatures were from other countries or regions, which do not adequately represent the overall population reactivation. Second, most of the recorded data were secondary extracts, due to which information such as age, gender, virus genotype, and occult infection could not be collected for further analysis. Here, we only determine whether a patient needs antiviral therapy based on the relapse rate of different drugs. However, the practical factors such as drug resistance, timing of prophylactic antiviral treatment discontinuation, and cost‐effectiveness should be also considered.

In summary, our meta‐analysis demonstrates a potential risk of HBV reactivation in HBsAg‐/HBcAb+ RA patients receiving RTX, especially HBsAb− patients. Our study furthers the understanding of the prophylactic use of anti‐HBV drugs in such patients. However, it is much safer to use inhibitors of IL‐6, TNF‐α, and JAK in these patients. Ultimately, doctor needs to consider all the patient's risk factors together and predict the level of risk of reactivation, then give an appropriate recommendation.

## AUTHOR CONTRIBUTIONS

**Xuezhi Hong**: Conceptualization (equal); Funding acquisition (supporting); Methodology (lead); Project administration (lead); Software (lead); Writing − review and editing (equal). **Yanhua Xiao**: Formal analysis (lead); Writing − original draft (lead); Writing − review and editing (equal). **Liyan Xu**: Data curation (equal); Investigation (equal); **Lei Liu**: Data curation (equal); Investigation (equal). **Hailu Mo**: Data curation (equal); Investigation (equal). **Hanyou Mo**: Conceptualization (equal); Funding acquisition (lead); Supervision (lead); Writing − review and editing (equal).

## CONFLICT OF INTEREST STATEMENT

The authors declare no conflict of interest.

## Supporting information

Supporting Information.

Supporting Information.
