# Supplementary figures and images for: Risk of hepatitis B reactivation in HBsAg−/HBcAb+ patients after biologic or JAK inhibitor therapy for rheumatoid arthritis: A meta‐analysis
Source: Immun Inflamm Dis. 2023 Feb 9;11(2):e780. doi: 10.1002/iid3.780 (PMC9910170; doi:10.1002/iid3.780)

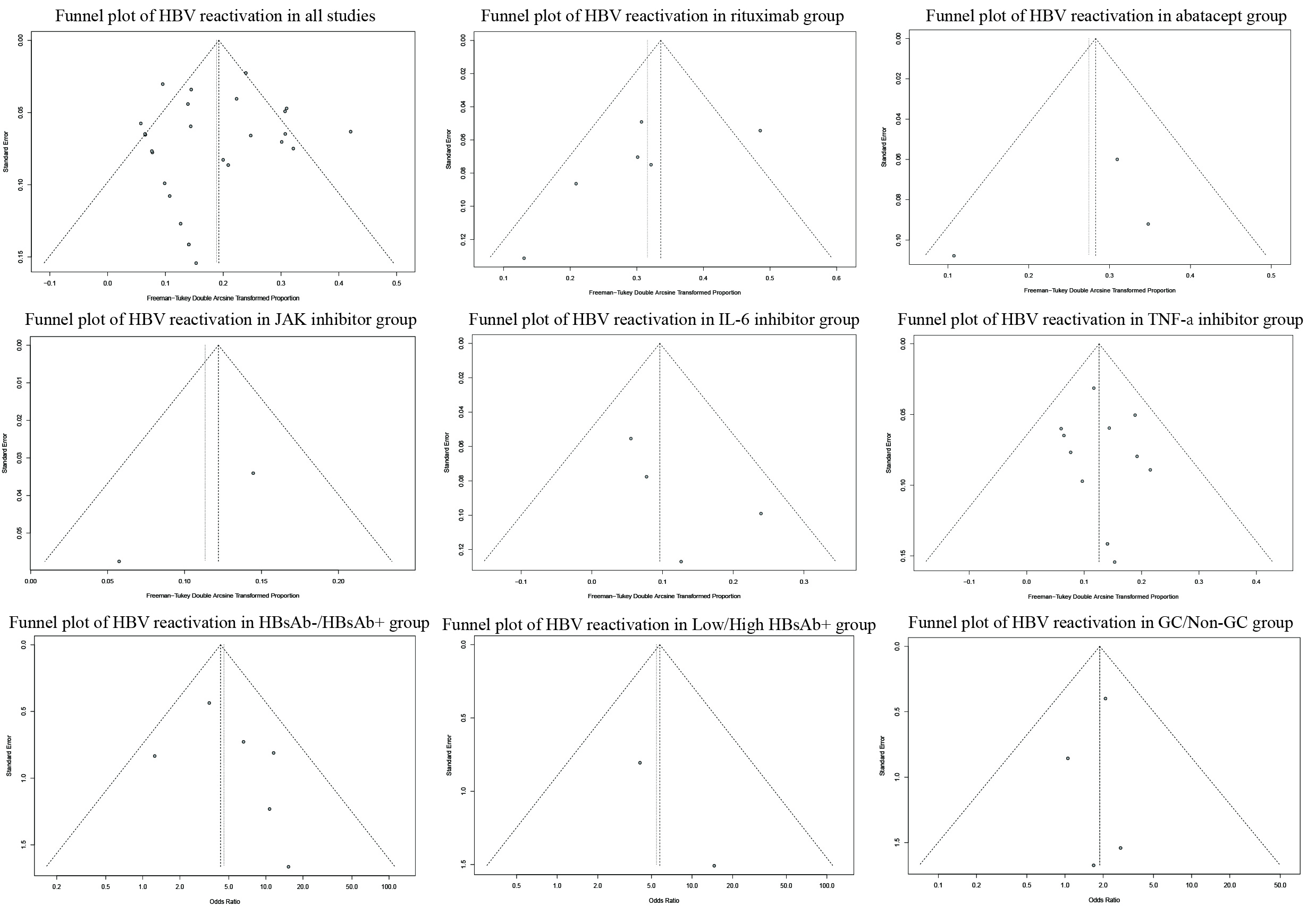

Supplement: Supplementary file 1 — Supporting Information. [file IID3-11-e780-s001.jpg]

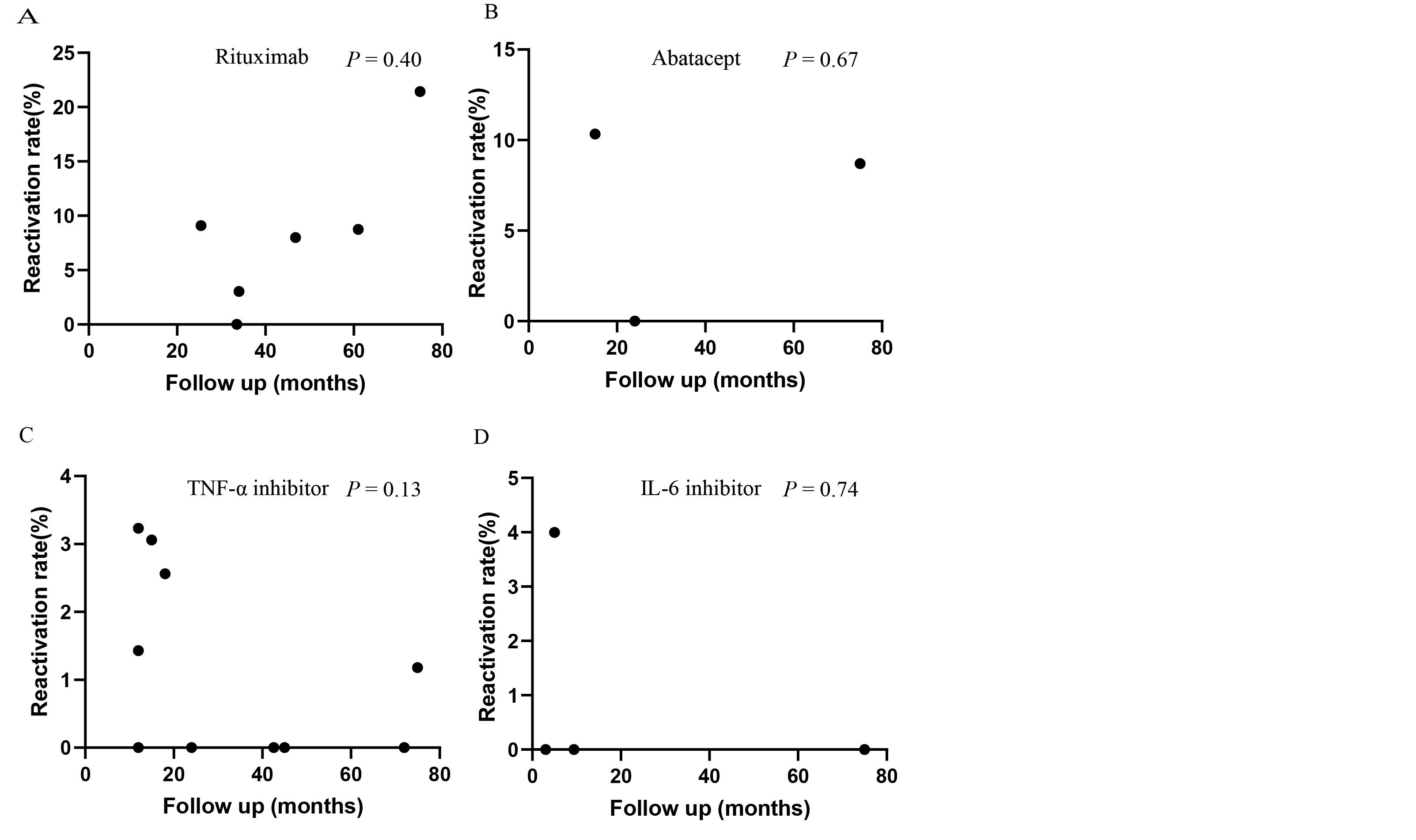

Supplement: Supplementary file 2 — Supporting Information. [file IID3-11-e780-s002.jpg]
